# Detection of subclinical cardiac involvement in inflammatory myopathy by CMR T1 relaxometry

**DOI:** 10.1186/1532-429X-18-S1-P254

**Published:** 2016-01-27

**Authors:** Marine Bravetti, Nadjia Kachenoura, Charles Roux, Marie Laure Chabi, Aude Rigolet, Olivier Benveniste, Philippe Cluzel, Alban Redheuil

**Affiliations:** 1 Cardiovascular Imaging, Cardiology Institute, Pitié Salpêtrière Hospital, Université Pierre et Marie Curie UPMC, Paris, France; 2 LIB Biomedical Imaging Laboratory INSERM and ICAN Institute of Cardiometabolism and Nutrition, Paris, France

## Background

While cardiac involvement including heart failure represents the first cause of mortality in idiopathic inflammatory myopathies (IM), subclinical alterations may be largely underdiagnosed by conventional tests. CMR T1 mapping may provide more sensitive diagnostic markers of cardiac involvement in both acute and chronic phases in IM irrespective of cardiac function or late gadolinium enhancement (LGE). Our aim was to determine the ability of CMR T1 relaxometry derived parameters to detect subclinical cardiac involvement in IM.

## Methods

Fifty-five patients (age 45 ± 13 years, 37 women) with proven IM were compared to 18 healthy controls comparable in age and body size. CMR was performed to evaluate biventricular function, LGE, T1 mapping using a 3(3)5 MOLLI sequence. Myocardial and blood T1 values were determined in a basal short axis view at baseline and 15 minutes after gadolinium injection and used to calculate T1 decay=100·[1-T1 post contrast/T1 baseline] and extra cellular volume (ECV). Biology included creatine kinase, troponin T and NT-proBNP.

## Results

Left and right end-diastolic ventricular volumes were smaller in patients compared to controls but ejection fractions were similar. Fourteen (25%) of the patients and none of the controls had LGE. T1 relaxometry parameters were significantly increased in patients compared to controls (T1 baseline: 1014 ± 46 ms vs. 985 ± 24 ms, p= 0.014; T1 decay: 71 ± 7% vs. 66 ± 5%, p= 0.001; ECV: 28 ± 3% vs. 16 ± 3%, p= 0.034). ROC area under curve for the diagnosis of IM were respectively for T1 baseline: 0.66; T1 decay: 0.81 and ECV: 0.73. T1 baseline values were positively related to increased BNP but not to troponin or CK levels. Eleven (20%) patients had LVEF<50%. Decreased LVEF was associated with increased LGE prevalence and T1 baseline values.

## Conclusions

Baseline myocardial T1, T1 decay and ECV were significantly increased in IM patients and demonstrated their ability to detect cardiac involvement irrespective of enzyme levels, even in patients with normal cardiac function and absence of LGE.

